# Future Morphology? Summary of Visual Word Identification Effects Draws Attention to Necessary Efforts in Understanding Morphological Processing

**DOI:** 10.3389/fpsyg.2012.00395

**Published:** 2012-10-09

**Authors:** Dirk Koester

**Affiliations:** ^1^Neurocognition and Action – Biomechanics Research Group, Faculty of Psychology and Sport Science, Bielefeld UniversityBielefeld, Germany; ^2^Center of Excellence – Cognitive Interaction Technology, Bielefeld UniversityBielefeld, Germany

This commentary discusses the insights and suggestions of a review by Amenta and Crepaldi (2012) in *Frontiers in Psychology*. The authors have diagnosed a controversial accumulation of findings in the field of visual word identification and, hence, have provided an overview of the field with the aim to separate substantial effects from findings that need further confirmation. The authors aim to provide a broad basis for theory development. Amenta and Crepaldi (2012) are the first to attempt a comprehensive psycholinguistic review of the major forms of word formation, namely inflection, derivation, and compounding. The authors summarize 17 robust experimental effects and suggest that “any theory should be able to explain” this set of experimental effects (cf. abstract). Thus, the listed effects are supposed to help to decide which among competing theories have more explanatory power and might thus be considered scientifically superior.

Without repeating all effects, Amenta and Crepaldi propose that stem frequency, family size, word entropy, and the number of affix allomorphs are main determinants in visual word identification. Furthermore, non-word processing is suggested to be relevant for morphological theories, if the non-words are made from morphemes. Other relevant properties are proposed for methodologically specified situations. For example, if stimuli are fully visible, morphological priming effects are only proposed for semantically related words, and inflectional priming yields greater effects than derivational priming. In contrast, in masked priming, morphological effects are comparable in magnitude for semantically transparent and opaque words. Also, inflectional and derivational priming is suggested to yield comparable effect sizes.

Amenta and Crepaldi’s review point toward relevant linguistic (e.g., productivity) and psycholinguistic variables (e.g., frequency measures) and their relations regarding visual word identification. Such knowledge will guide future investigations and, hence, impact also models of language performance. The authors suggest that these findings provide a basis for the evaluation of competing theories and, in doing so, to contribute to future theory development; in their own words, to construct an “all-inclusive model of visual identification of morphologically complex words.” In light of the specificity of the insights, these broad suggestions leave the reader with the impression of a gap between insights and suggestions. The authors deal with a specific functional step (visual word identification) of the more complex human ability of (single word) reading and it is not necessary that the relevant variables for identification can be extrapolated to other functional steps (e.g., morphosyntactic and/or semantic combination of morpho-orthographic segments).

As the authors mentioned themselves, their list of effects is not exhaustive. However, one would like to know whether, and if so, what role further variables such as surface frequency, word length, word class, abstractness, or cues to morpheme boundaries are supposed to play in word identification (e.g., Caramazza and Hillis, 1991; Inhoff et al., 2000; Taft, 2004; Baayen et al., 2007; Juhasz, 2008; Kuperman et al., 2009; Juhasz and Pollatsek, 2011; Hyönä, 2012). The impact of Amenta and Crepaldi’s (2012) target list of relevant effects on future experimentation and theory development will depend on the relative contribution of all these variables. Consequently, one needs to discuss whether the additional variables mentioned here affect only later reading stages or what their role could be during word identification.

More generally, the authors seem to aim for a psycholinguistic, i.e., cognitive model of language behavior rather than a linguistic theory. (This is not the same; morphological effects can, for example, be simulated without an implementation of morphology; cf. Baayen et al., 2011.) They also refer to some eye-tracking and electrophysiological studies which provide neural evidence. It remains unclear how the neural evidence is to be incorporated into a strictly cognitive model. Alternatively, one may aim for a neuro-cognitive model of language behavior and visual word identification in particular. If one is to construct a complete model of such a phenomenon, the effects (behavior) but also the causes (neural activity) appear to be relevant and should be considered. While Amemta and Crepaldi’s (2012) work to consider the large body of behavioral evidence is certainly ambitious, future work should take neural evidence also into account because cognitive and neural evidence can be mutually informative and helpful in understanding language performance (Grimaldi, 2012).

Another methodological issue arises from the suggestion that models of visual word identification should explain the effects listed by Amenta and Crepaldi (2012) because the list comprises aspects of experimental techniques (masking). Masking does not pertain to the phenomenon in question but experimental paradigms can be modeled. For example, Norris and Kinoshita (2008) proposed that in masked priming, prime, and target are perceptually fused into a single percept or object. As a consequence, masked priming effects may depend on the task requirements rather than on the relation between prime and target representations. Norris and Kinoshita (2008) show that priming effects can be shifted from word stimuli to non-word stimuli by using a same-different task rather than a lexical decision task. For the present discussion, one can doubt whether psycholinguistic models of word identification have to combine methodological aspects such as masked priming with the processes of interest, i.e., visual word identification.

Although some questions remain, Amenta and Crepaldi’s (2012) review is bound to stimulate scientific discussions regarding visual word identification and provoke further research efforts to better understand morphological processing. Next steps of enquiry may focus on the different domains of morphology, inflection, derivation, or compounding drawing on as many sources of evidence as possible (e.g., different populations, methodologies, or language families) to comprehensively describe each domain (cf. Niemi et al., 1994; Marslen-Wilson and Tyler, 2007). Another major challenge is the theoretical unification of different sensory modalities. Finally, future reviews would be highly informative, if they use quantitative evaluations of research findings. One might perform meta-analyses or quantify the frequency of replications of particular effects. This way, our understanding of the connection between morphology and how it is represented and controlled by the human brain may be fostered (cf. Grimaldi, 2012).

## References

[B1] Amenta S. Crepaldi D. (2012). Morphological processing as we know it: an analytical review of morphological effects in visual word identification. Front. Psychol. 3:232 10.3389/fpsyg.2012.00232 22807919 PMC3395049

[B2] Baayen R. H. Milin P. Ðurđević D. F. Hendrix P. Marelli M. (2011). An amorphous model for morphological processing in visual comprehension based on naive discriminative learning. Psychol. Rev. 118, 438–482 10.1037/a0023851 21744979

[B3] Baayen R. H. Wurm L. H. Aycock J. (2007). Lexical dynamics for low frequency complex words: a regression study across tasks and modalities. Ment. Lex. 2, 419–463

[B4] Caramazza A. Hillis A. E. (1991). Lexical organization of nouns and verbs in the brain. Nature 349, 788–790 10.1038/349788a0 2000148

[B5] Grimaldi M. (2012). Toward a neural theory of language: old issues and new perspectives. J. Neurolinguistics 25, 304–327 10.1016/j.jneuroling.2012.02.001

[B6] Hyönä J. (2012). The role of visual acuity and segmentation cues in compound word identification. Front. Psychol. 3:188 10.3389/fpsyg.2012.00188 22701444 PMC3371694

[B7] Inhoff A. W. Radach R. Heller D. (2000). Complex compounds in German: interword spaces facilitate segmentation but hinder assignment of meaning. J. Mem. Lang. 42, 23–50 10.1006/jmla.1999.2666

[B8] Juhasz B. (2008). The processing of compound words in English: effects of word length on eye movements during reading. Lang. Cogn. Process. 23, 1057–1088 10.1080/01690960802144434

[B9] Juhasz B. Pollatsek A. (2011). “Lexical influences on eye movements in reading,” in The Oxford Handbook of Eye Movements, eds Liversedge S. Gilchrist I. Everling S. (Oxford: Oxford University Press), 873–893

[B10] Kuperman V. Schreuder R. Bertram R. Baayen R. H. (2009). Reading polymorphemic Dutch compounds: toward a multiple route model of lexical processing. J. Exp. Psychol. Hum. Percept. Perform. 35, 876–895 10.1037/a0013484 19485697

[B11] Marslen-Wilson W. Tyler K. K. (2007). Morphology, language and the brain: the decompositional substrate for language comprehension. Philos. Trans. R. Soc. Lond. B Biol. Sci. 362, 823–836 10.1098/rstb.2007.2091 17395577 PMC2430000

[B12] Niemi J. Laine M. Tuominen J. (1994). Cognitive morphology in Finnish: foundations of a new model. Lang. Cogn. Process. 9, 423–446 10.1080/01690969408402126

[B13] Norris D. Kinoshita S. (2008). Perception as evidence accumulation and Bayesian inference. Insights from masked priming. J. Exp. Psychol. Gen. 137, 434–455 10.1037/a0012799 18729709

[B14] Taft M. (2004). Morphological decomposition and the reverse base frequency effect. Q. J. Exp. Psychol. A 57, 745–765 10.1080/02724980343000477 15204131

